# Supplementary material for: Genomic Footprints of Historical Introgression Between Ancient Lineages of Wild Oryza AA-Genome Species With Widely Separated Contemporary Distributions
Source: Genome Biol Evol. 2026 Sep 5;18(9):evag191. doi: 10.1093/gbe/evag191 (PMC13545100; doi:10.1093/gbe/evag191)
Supplement: Supplementary file 1 [file evag191_supplementary_data.pdf]

Supplementary Materials

Supplementary Figure 1. Phylogenetic networks inferred with PhyloNet. (a) Total log probabilities of inferred networks across 10 independent runs are shown for each maximum number of reticulation events (0–10). Red circles indicate the mean and the error bars indicate the standard deviation. (b) The maximum likelihood network allowing five reticulation events. Dashed lines represent putative hybridization events.

Supplementary Figure 2. Estimated divergence time of AA-genome species. Numbers on the nodes represent time in million years ago (MYA). Blue bars indicate the 95% highest posterior density (HPD) intervals.

Supplementary Figure 3. Genomic distribution of candidate introgressed regions. The plots show the genomic location of alignment blocks (bootstrap support $\geq$ 90) where the nearest neighbors of (a) *O. meridionalis* and (b) *O. longistaminata* are classified into the four categories (Africa, America, Asia, and 'Other').

(a)

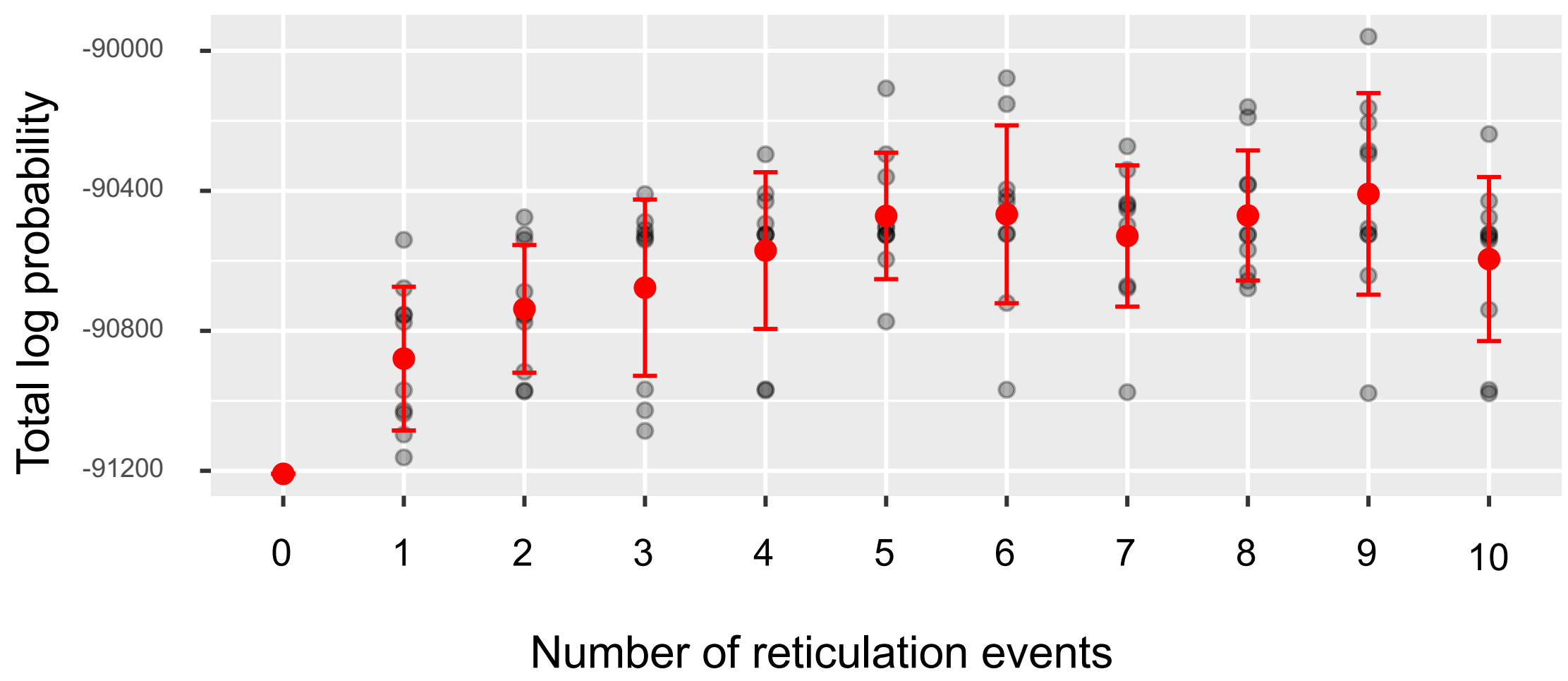

(b)

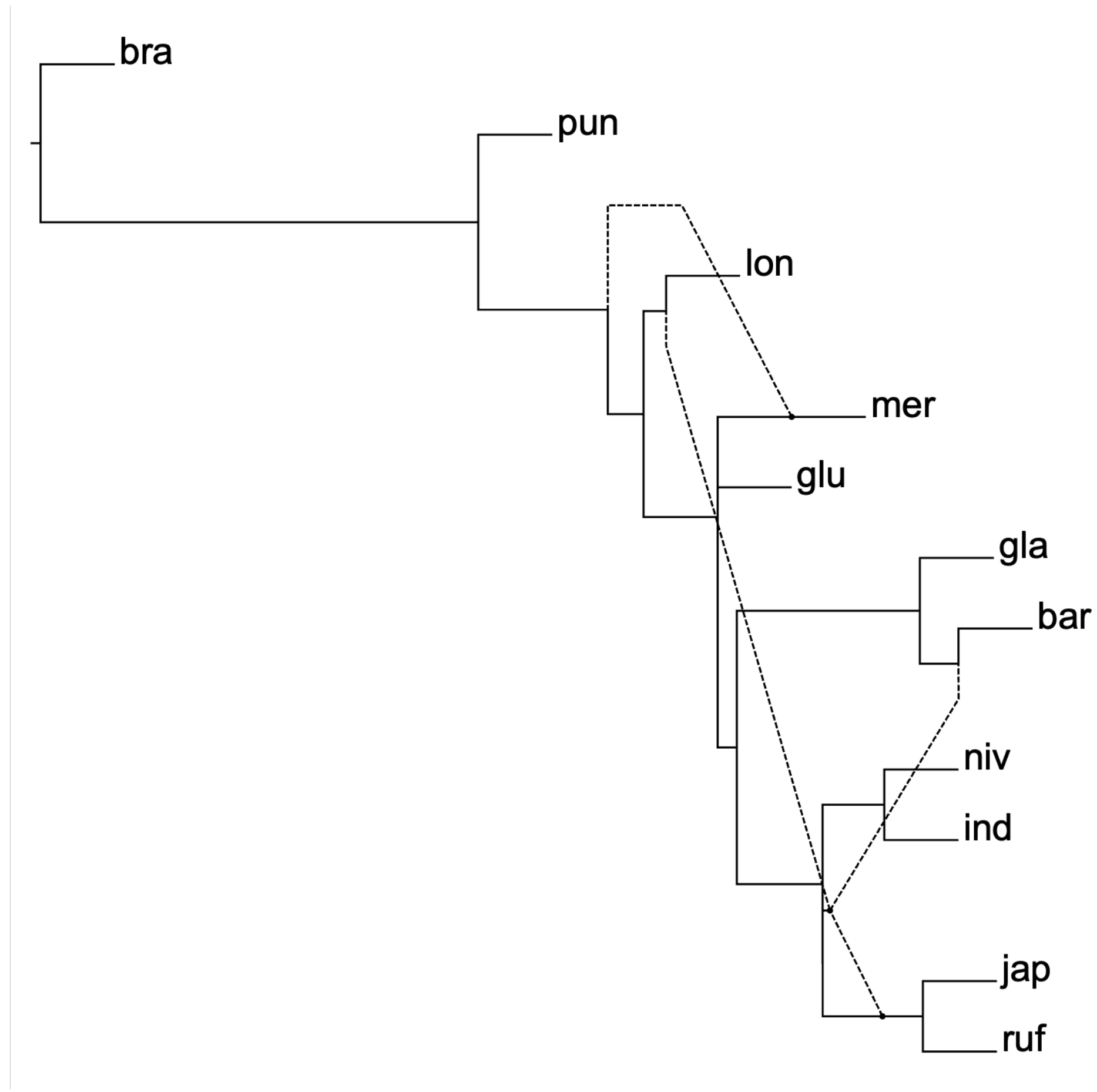

Supplementary Fig. 1

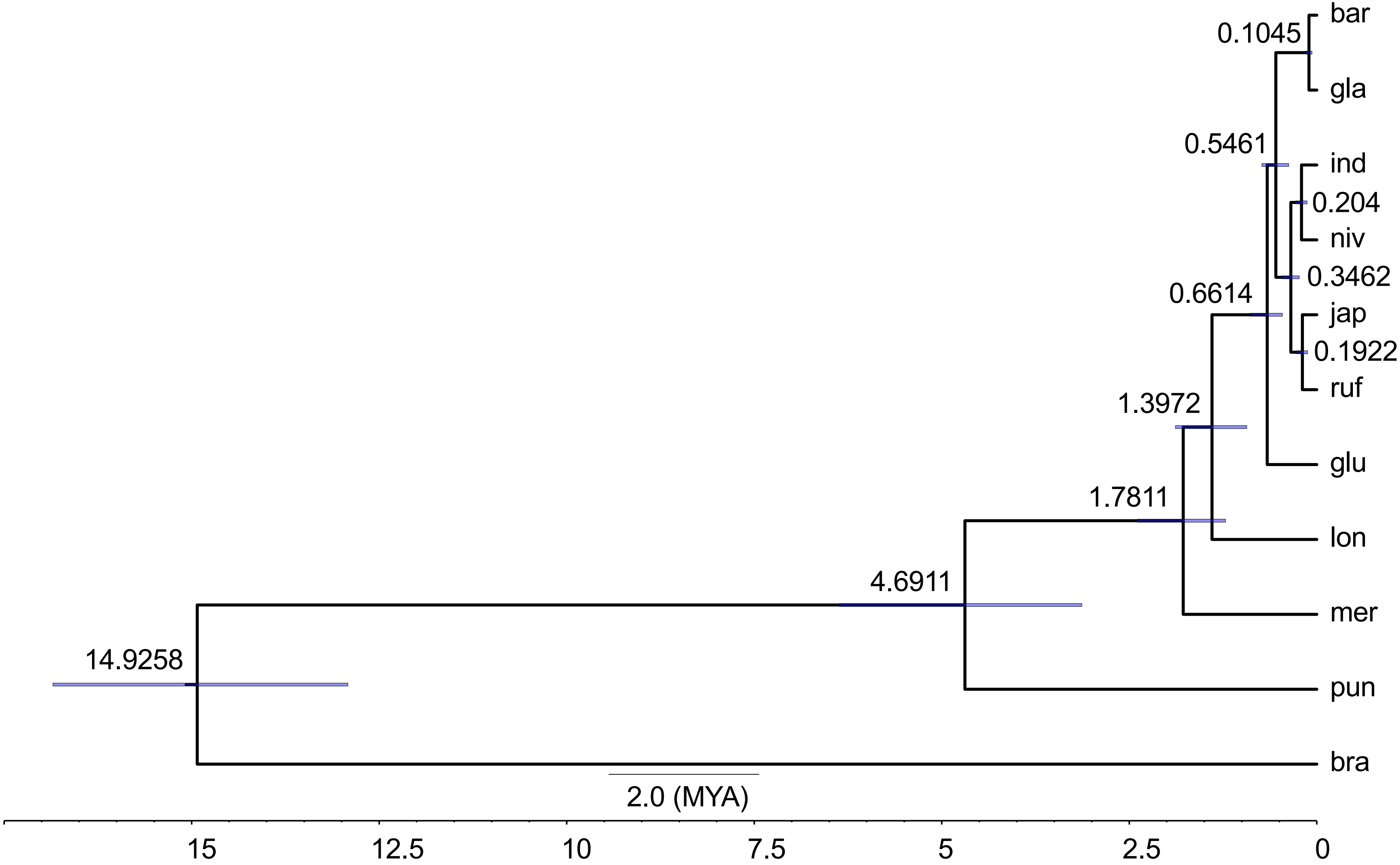

Supplementary Fig. 2

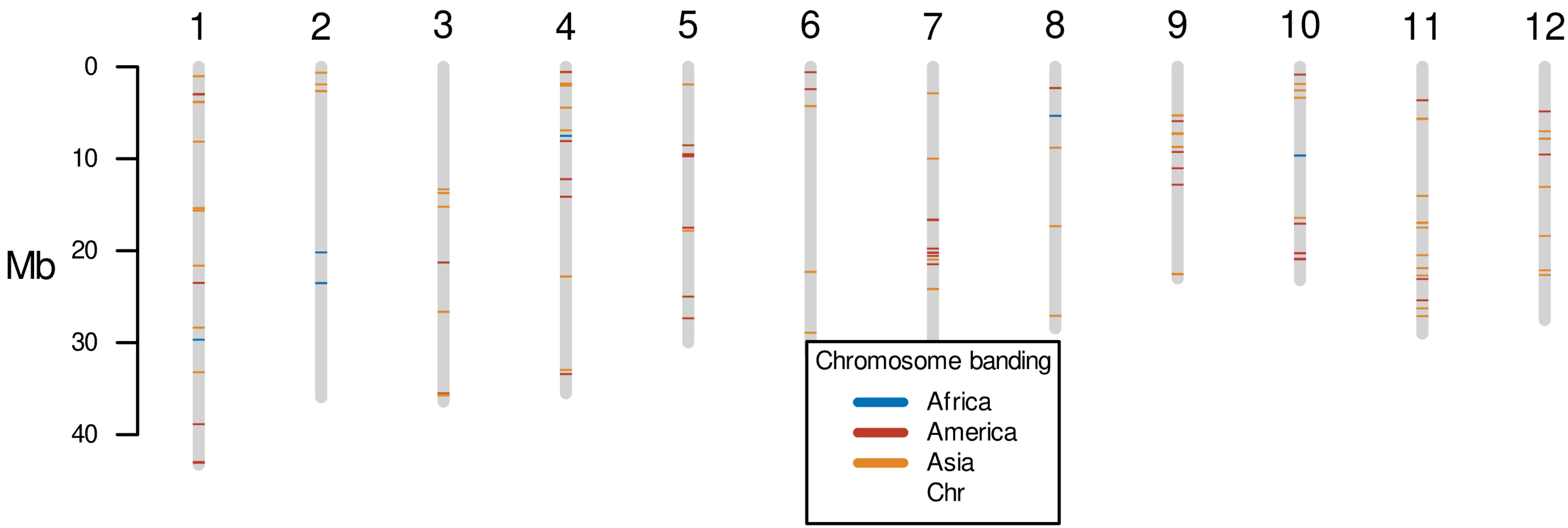

O. meridionalis

Supplementary Fig. 3a

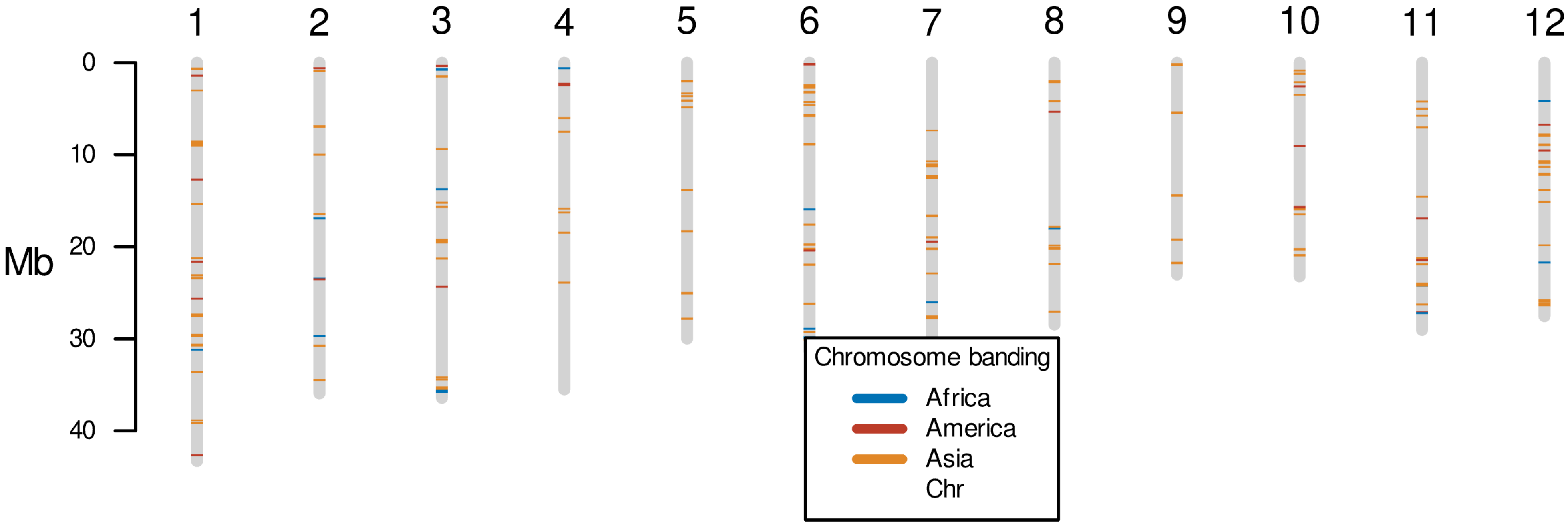

O. longistaminata

Supplementary Fig. 3b
